# Tree species identity determines wood decomposition via microclimatic effects

**DOI:** 10.1002/ece3.5665

**Published:** 2019-09-27

**Authors:** Felix Gottschall, Sophie Davids, Till E. Newiger‐Dous, Harald Auge, Simone Cesarz, Nico Eisenhauer

**Affiliations:** ^1^ German Centre for Integrative Biodiversity Research (iDiv) Halle‐Jena‐Leipzig Leipzig Germany; ^2^ Institute of Biology Leipzig University Leipzig Germany; ^3^ Department of Community Ecology Helmholtz‐Centre for Environmental Research – UFZ Halle Germany

**Keywords:** aboveground–belowground interactions, biodiversity–ecosystem functioning, soil microbial properties, temperature, tree species richness, wood mass loss

## Abstract

Empirical evidence suggests that the rich set of ecosystem functions and nature's contributions to people provided by forests depends on tree diversity. Biodiversity–ecosystem functioning research revealed that not only species richness per se but also other facets of tree diversity, such as tree identity, have to be considered to understand the underlying mechanisms. One important ecosystem function in forests is the decomposition of deadwood that plays a vital role in carbon and nutrient cycling and is assumed to be determined by above‐ and belowground interactions. However, the actual influence of tree diversity on wood decay in forests remains inconclusive. Recent studies suggest an important role of microclimate and advocate a systematical consideration of small‐scale environmental conditions. We studied the influence of tree species richness, tree species identity, and microclimatic conditions on wood decomposition in a 12‐year‐old tree diversity experiment in Germany, containing six native species within a tree species richness gradient. We assessed wood mass loss, soil microbial properties, and soil surface temperature in high temporal resolution. Our study shows a significant influence of tree species identity on all three variables. The presence of Scots pine strongly increased wood mass loss, while the presence of Norway spruce decreased it. This could be attributed to structural differences in the litter layer that were modifying the capability of plots to hold the soil surface temperature at night, consequently leading to enhanced decomposition rates in plots with higher nighttime surface temperatures. Therefore, our study confirmed the critical role of microclimate for wood decomposition in forests and showed that soil microbial properties alone were not sufficient to predict wood decay. We conclude that tree diversity effects on ecosystem functions may include different biodiversity facets, such as tree identity, tree traits, and functional and structural diversity, in influencing the abiotic and biotic soil properties.

## Graphical abstract

We studied the influence of tree species richness, tree species identity, soil microbial properties, and microclimatic conditions on wood decomposition in a temperate tree diversity experiment. We found significant tree species identity effects on wood decomposition due to modified nighttime soil surface temperatures. Our study confirmed the critical role of microclimate for wood decomposition in forests and showed that soil microbial properties alone were not sufficient to predict wood decay.

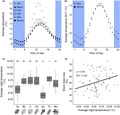

## 1 INTRODUCTION

Forest ecosystems provide a rich set of ecosystem functions contributing to human well‐being (Díaz et al., 2018; Nadrowski, Wirth, & Scherer‐Lorenzen, 2010; Scherer‐Lorenzen, Schulze, Don, Schumacher, & Weller, 2007). Based on an ever‐increasing number of studies over the last decade (Gamfeldt et al., 2013; Huang et al., 2018; Paquette & Messier, 2011; Tobner et al., 2016), there is strong empirical evidence for a positive relationship between tree diversity and ecosystem functions, such as biomass production or nutrient cycling, which have implications for a rich set of nature's contributions to people (Díaz et al., 2018; Gamfeldt et al., 2013; Nadrowski et al., 2010). This work emphasizes the repeatedly stated relevance of biodiversity for the functioning and service supply of ecosystems in general (Cardinale et al., 2012; Díaz et al., 2018; Millenium Ecosystem Assessment, 2005; Rockström et al., 2009) and further underlines the importance of biodiversity–ecosystem functioning research in forest ecosystems (Bruelheide et al., 2014; Eisenhauer et al., 2016; Verheyen et al., 2016).

Biodiversity–ecosystem functioning research has shown that not only species richness per se but also other facets of biodiversity, such as trait identity and diversity reflecting functional differences among species, have to be considered to understand biodiversity effects and to reveal the underlying mechanisms (Craven et al., 2018; Ebeling et al., 2014; Eisenhauer et al., 2016; Scherer‐Lorenzen, Bonilla, & Potvin, 2007; Schuldt et al., 2019). A high diversity of functional traits is likely to increase resource use efficiency through niche partitioning and resource use complementarity (Hillebrand, Bennett, & Cadotte, 2008). However, it is also possible that a single species and certain traits (Roscher et al., 2012) dominate a community (selection effect) and its functioning due to its generally higher productivity or adaptation to environmental factors and tree stand conditions (Tobner et al., 2016).

Many ecosystem functions substantially depend on soil processes facilitated by above‐ and belowground linkages (Wall, Bardgett, & Kelly, 2010; Wardle et al., 2004). Through the input of leaf litter, root litter, and root exudates, trees influence the resource availability for soil food webs (Cesarz et al., 2013; Prescott, 2002; Schwarz et al., 2015). The chemical and physical properties of litter from different trees differ substantially (Augusto, Ranger, Binkley, & Rothe, 2002; Grayston, Vaughan, & Jones, 1997) and therefore affect soil detrital food webs in several ways. A more diverse plant community is characterized by a more diverse composition of different litter substrates, determining resource availability for soil microorganisms, which can lead to cascading effects on the diversity and functioning of soil microorganisms, as well as whole food webs (Cesarz et al., 2013; Hooper et al., 2000; Milcu, Partsch, Langel, & Scheu, 2006; Wardle, Yeates, Barker, & Bonner, 2006). Thus, these aboveground–belowground biodiversity effects have a major impact on ecosystem processes, such as decomposition, nutrient cycles, and plant biomass production (Bardgett & Van Der Putten, 2014; Cardinale et al., 2011; Gessner et al., 2010; Hooper et al., 2000; Prescott, 2002; Wardle et al., 2004).

The decomposition of deadwood plays a vital role for carbon and nutrient cycling in forest ecosystems worldwide (Chao et al., 2009; Cornwell et al., 2009; Delaney, Brown, Lugo, Torres‐Lezama, & Quintero, 1998). However, the influence of tree species richness on wood decay in forests remains inconclusive (Gessner et al., 2010; Pietsch et al., 2018; Scherer‐Lorenzen, Bonilla, et al., 2007). Decomposition is mainly driven by microbial activity (bacteria and fungi), which again is strongly dependent on substrate quality, soil chemical properties, soil temperature, soil moisture, and decomposer fauna (Cornwell et al., 2009; Hattenschwiler, Tiunov, & Scheu, 2005). Accordingly, there are two main groups of mechanisms that may link tree diversity and decomposition. First, tree species diversity can alter decomposition rates *via* species‐specific traits related to the quality of the dead organic substrates, such as leaf litter and wood. This resource quality is then expected to drive the biomass, activity, and diversity of microorganisms that can determine decomposition through complementarity or selection effects (Gartner & Cardon, 2004; Gessner et al., 2010; Handa et al., 2014; Hattenschwiler et al., 2005). Second, there is evidence for environmental changes caused by tree diversity and identity, including alterations of soil pH, moisture, and temperature, which are significant determinants of soil microbial community composition and activity, and subsequently of decomposition (Hattenschwiler et al., 2005; Joly et al., 2017; Pietsch et al., 2018). For instance, Joly et al. (2017) showed that tree species composition can alter microenvironmental conditions to an extent that overrides the impact of macroclimate on ecosystem functions. Accordingly, they recommended to consider microclimatic conditions in future studies of tree community effects on decomposition.

To investigate the role of tree species richness and tree species identity on wood decomposition via soil microbial communities and microclimate, we conducted a field experiment in a 12‐year‐old tree diversity experiment (with tree monocultures and 2‐, 3‐, and 5‐species mixtures) in Central Germany. To explore the underlying mechanisms of potential tree species richness and identity effects, we investigated soil basal respiration and soil microbial biomass and assessed soil surface temperatures in high temporal resolution. We hypothesized that (i) tree species richness will increase wood decomposition, while (ii) tree species identity effects on wood decomposition will depend on litter quality traits: Tree species with low C:N litter will increase wood decomposition, while tree species with high C:N litter will decrease wood decomposition. Furthermore, we hypothesized that decomposition will be higher with (iii) increased soil microbial biomass and activity (Gessner et al., 2010) as well as under (iv) increased average soil surface temperatures (Joly et al., 2017).

## 2 MATERIAL AND METHODS

### 2.1 Study site

**Figure 1:**
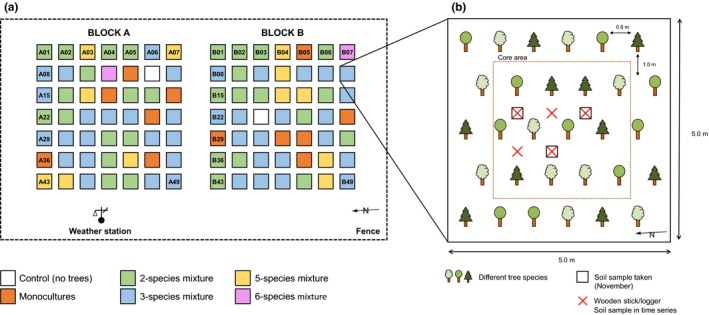
(a) Top view on experimental design and (b) plot chart. Redrawn after (Hantsch et al., 2014). Numbers indicate plot numbers. The defined subplots are congruent with the red crosses. The dotted line represents the core area that was established to reduce edge effects

The Kreinitz Tree Diversity experiment (51°23′10″N, 13°15′43″E) was set up on a former arable field in Germany (managed until 1990, and abandoned thereafter) in 2005 (Hantsch et al., 2014). The site has a slightly acidic soil (pH 4.6–6.3) with a sandy texture and no considerable slope. The experiment is divided into two blocks (A and B), each of them covering 49 plots (25 m^2^ each) randomly assigned to the diversity levels and species combinations described below (Figure 1a). On each plot, except the control plots, 30 randomly arranged tree saplings (2 years old) were planted in five rows with six saplings per row. The distance in between two rows is 1 m; the distance among trees within a row is 0.8 m (Figure 1b). Each plot contains a core area of 3 m x 3 m comprising the 12 inner tree individuals to prevent edge effects. The community composition of the core areas matches the composition of the respective plot. The species pool of the experiment consists of six native Central European tree species: *Fagus sylvatica* (European beech; abbreviated as “Be”), *Fraxinus excelsior* (Common ash; “As”), *Picea abies* (Norway spruce; “Sp”), *Pinus sylvestris* (Scots pine; “Pi”), *Tilia cordata* (Small‐leaved lime; “Li”), and *Quercus petraea* (Sessile oak; “Oa”). Within one block, the tree species richness gradient reaches from plots without any trees (*n* = 1 plot; control plot), monocultures of each species (*n* = 6 plots), every possible combination of two species (*n* = 15 plots), every possible combination of three species (*n* = 20 plots), every possible combination of five species (*n* = 6 plots) to six species (*n* = 1 plot). Since the control plots do not contain trees, they were not used in this study. Furthermore, we did not use the data from the six species combination, since they contain all tree species of the experiment, making the analysis of tree identity on those plots inconclusive.

### 2.2 Soil sampling and processing

In November 2017 (i.e., 12 years after the setup of the experiment), soil from each plot (except the control plots) was sampled to a depth of 5 cm using cylindrical steel soil corers with a diameter of 5 cm. The wide diameter and shallow depth were chosen to maximize the amount of soil that was directly in contact with the wooden sticks or close to the soil surface. To account for spatial heterogeneity, three soil cores per plot were taken on defined positions (Figure 1b) and pooled in the field, resulting in 96 soil samples in total. The litter layer was removed before sampling. During the sampling event, the soil samples were cooled and later transferred to a 4°C fridge for 4 days until further processing. Subsequent to the sampling, we sieved all samples at 2 mm to homogenize the soil and remove stones, roots, and large soil animals. The samples were used to determine soil microbial respiration and biomass at the end of the decomposition period.

To study more general relationships between tree diversity and soil ecosystem functions, additional samples were taken every two months from August 2016 to October 2017 using a steel soil corer (2.5 cm diameter, standard depth of 10 cm after removing the litter layer). Samples were taken on a subset of the tree diversity gradient, that is, all monoculture and 5‐species mixture plots on five defined subplots (Figure 1b). The soil was processed and analyzed in the same way as the soil taken in November 2017. The samples were used to determine an integrated measure of soil microbial biomass across the study period, excluding possible artifacts of snapshot measurements.

### 2.3 Leaf litter collection and carbon‐to‐nitrogen ratio

In parallel to the soil sampling in November 2017, we randomly collected approximately 30 g of leaf litter material out of the core area on all monoculture and 5‐species mixture plots (Figure 1a). The samples were stored at 4°C in the laboratory. For further processing, we sorted the leaf litter of each sample according to their species. Afterward, they were dried at a constant temperature of 40°C for 72 hr. All samples were ground in a ball mill (MM2000; Retsch GmbH). To check for differences in carbon‐to‐nitrogen ratio (C:N) of leaf litter of different treatments, we analyzed 40 mg of ground material by dry combustion (Vario EL cube; Elementar Analysensysteme GmbH). We added 40 mg of Tungsten(VI)‐oxide (ratio 1:1) and purged and trapped CO_2_ as well as SO_2_ using a Thermal Conductivity Detector.

### 2.4 Wood mass loss measurements

In June 2016, we placed five wooden sticks (tongue depressors, NOBA Verbandsmittel GmbH u. Co. KG, D‐58300 Wetter, *Betula* spec.) between the soil and litter layer at five defined positions within each plot (Figure 1b). Before placing the sticks on the plots, we oven‐dried them at 70°C for 48 hr to remove any water content. After drying and cooling down to constant weight, each stick was weighted and labeled with a unique ID to account for weight deviation caused by the manufacturing process. During the exposure period (from June 2016 to November 2017), we regularly assessed the condition of the sticks to prevent overdecomposition. For this, we carefully uncovered a random subset of sticks and estimated the mass loss directly on the plots. After this assessment, the sticks were covered again. In November 2017 (after 18 months), we collected the sticks. Out of 480 sticks initially deployed in the plots, 372 could be retrieved. We assume that animal and regular scientific activities on the plots might have caused the loss and damage of some of the sticks that could not be evaluated. In the laboratory, the sticks were carefully cleaned from soil using water. To prevent wood material loss, a sieve was put underneath the wood sticks. After washing, the wooden sticks were dried at 40°C for 72 hr to constant weight, removing any residual water.

Wood mass loss as a measure of wood decomposition was calculated as the percent of missing dry weight after exposure compared to the start dry weight before exposure. In case of clearly broken off and missing (not decomposed) wood pieces, we extrapolated the weight of the stick *via* the lost surface area. To do so, we created a stick template on millimeter paper to determine the surface area broken off by counting missing mm^2^. This area was used to extrapolate the total weight based on the remaining dry weight of the recovered stick. For the final analysis, we only used clearly undamaged sticks and sticks which only lost <50% of their area through breaking. Accordingly, 77% of all sticks brought to the field were used for the analysis, and these covered all the experimental plots with multiple sticks per plot.

### 2.5 Microbial biomass and activity

To investigate the activity and biomass of soil microorganisms, we used an automated electrolytic microrespirometer (Scheu, 1992). In a first step, we measured soil basal respiration (BR) to assess soil microbial activity (µl O_2_ hr^−1^ g^−1^ soil dry weight). For this purpose, 6 g (fresh weight) of soil per sample was used without the addition of any substrate. In a second step, we used the same soil to measure the maximal initial respiratory response (MIRR) to a single addition of a defined amount of glucose (0.008 g d‐glucose g^−1^ soil dry weight in 1.5 ml distilled water) to determine soil microbial biomass (µg C_mic_ g^−1^ soil dry weight) by calculating MIRR × 38 according to Beck et al. (1997). The soil samples taken in November 2017 and the soil samples from the time series (August 2016–October 2017) were treated the same way.

### 2.6 Soil surface temperature

The soil surface temperature was measured on the subplot level (Figure 1b) using temperature loggers (HOBO Pendant® Temperature/Light 8K Data Logger, Onset Computer Corporation®) between the soil and litter layer of monocultures and 5‐species mixtures. Thus, the loggers were exposed to the same conditions as the wooden sticks during the decomposition period. Temperature was logged every 30 min for 11 months (February 2017–January 2018).

### 2.7 Data analysis

For all statistical analyses, we used the R software, v.3.5.1 (R Core Team, 2018), and the *lme4* package (Bates, Mächler, Bolker, & Walker, 2015) to fit linear mixed‐effects models. For all models, an analysis of variance (ANOVA) was performed. The package *ggplot2* (Wickham, 2016) was used for data visualization.

### 2.8 Tree species richness and identity effects

Linear mixed‐effects models were used to test the effects of tree species identity (presence/absence of a certain tree species within a plot) and tree species richness (TSR; as continuous variable) on wood decomposition (i.e., wood mass loss, WML), soil BR, and soil microbial biomass (C_mic_). All models included the experimental blocks as random effect. Models testing for tree species richness also included the plots' different tree compositions (*n* = 48) nested in tree species richness as random effect. For microbial biomass and basal respiration, we added the machine ID (M_ID_) of different respirometers as another random effect to account for possible differences among measuring devices. For tree identity, it was not possible to include the presence of all six tree species simultaneously in the final models due to model saturation. Thus, final models only included those tree species that showed a significant effect on the respective response variable, within separate models for each individual tree species that were tested beforehand. The final model also considered interactions between the presence of species and tree species richness (see formulas (1), (2), (3) below).

Akaike information criterion (AIC) was used to estimate the relative quality of all final models (based on ∆AIC in between two models, where ∆AIC had to be > 2). Although using model selection under a given experimental design has been criticized (Colegrave & Ruxton, 2017; Hurlbert, 2009), this approach was chosen here, given that the presence of a certain tree species and interaction effects with other tree species (i.e., an important aspect of our analyses) were not completely balanced in the experimental design. As a result of the species selection process and the model selection based on ∆AIC, we modeled the three ecosystem functions using the following R syntax:

(1) $$\begin{array}{cc} \text{WML} & ∼\text{TSR}+\text{Presence of spruce}+\text{Presence of pine}\\ & \;+\text{TSR: Presence of spruce}+\text{TSR:Presence of pine}\\ & \;+\text{Presence of spruce:Presence of pine}+\left(1|\text{Experimental block}\right)\\ & \;+(1|\text{TSR/Species composition)} \end{array}$$

(2) $$\begin{array}{cc} \text{BR} & ∼\text{TSR}+\text{Presence of spruce}+\text{Presence of pine}+\text{TSR:Presence of pine}\\ & \;+\text{Presence of spruce:Presence of pine}+\left(1|\text{Experimental block}\right)\\ & \;+\left(\text{1|TSR/Species composition}\right)+(1|{\;M}_{\text{ID}}) \end{array}$$

(3) $$\begin{array}{cc} C_{\text{mic}} & ∼\text{TSR}+\text{Presence of spruce}+\text{Presence of pine}+\text{Presence of beech}\\ & \;+\text{Presence of oak}+\text{TSR:Presence of beech}\\ & \;+\text{Presence of pine:Presence of beech}+\text{Presence of pine}\\ & \;+\text{Presence of oak}+\text{Presence of spruce}+\text{Presence of oak}\\ & \;+\left(1|\text{Experimental block}\right)+\left(1|\text{TSR/Species composition}\right)+(1\left|M_{\text{ID}}\right) \end{array}$$

Tree identity effects on C:N ration were tested with a linear mixed‐effects model including plot number nested in block as independent random effects. The function *plot_model()* of the R package *sjPlot* (Lüdecke, 2016) was used to plot effect sizes.

### 2.9 Tree identity effects on integrated soil microbial biomass over time

We further tested tree identity effects on the temporal average soil microbial biomass in monocultures and 5‐species mixtures (taken every 2 months between August 2016 and October 2017). To test for identity effects, we used a linear mixed‐effects model, using plot identity (species identity of monoculture or 5‐species mixture) nested in experimental block, nested in sampling event (month of sampling) as a random factor. We considered the repeated measurements within the plots by adding an autoregressive structure to the random effect. Therefore, we evaluated compound symmetry covariance and first‐order autoregressive structures based on the AIC. With ΔAIC < 2, the model including the simplest covariance structure (i.e., compound symmetry) was chosen. We then performed Tukey's range test to determine differences between the plot identities using the *multcomp* package (Hothorn, Bretz, & Westfall, 2008).

### 2.10 Effects of integrated soil microbial biomass on wood mass loss

We tested the effect of average soil microbial biomass on monocultures and 5‐species mixtures during the exposure period (time series data from August 2016 to October 2017) on wood mass loss. For this, we used a linear mixed‐effects model, including plot number nested in block, as well as the plot identity (As, Be, Li, Oa, Pi, Sp, and 5‐species mixture) incorporated as a random effect. The reader should note though that this test cannot infer causality, although basing on the assumption that wood decomposition would increase with increasing soil microbial biomass.

### 2.11 Average night soil surface temperature and its influence on wood mass loss

In addition to the average soil surface temperature during 24 hr (over the whole measurement period), the average night temperature per calendar day during the exposure period was calculated to exclude the heating effect of direct sunlight on spots without shading. Therefore, we used the temperature data between 10 p.m. (CET) and 6 a.m. (CET) to capture a stable timeframe without sunlight throughout the year. Moving the timeframe by ± 2 hr did not change the results over seasons, indicating that the chosen timeframe was robust over the whole year. The effect of tree identity on average night soil surface temperature was tested using a linear mixed‐effects model. We included plot number nested in experimental block as a random‐effects term.

To test the influence of the average night temperature on wood mass loss, the subset of the available wood decomposition data on subplot level was used (i.e., five samples and loggers per plot, including only monocultures and five species mixtures) to match the available temperature data. The model included plot number nested in block as well as plot identity (As, Be, Li, Oa, Pi, Sp, and 5‐species mixture) as random effects.

### 2.12 Seasonal effect on microbial biomass

To test the influence of tree phenology (i.e., reduced overall activity of deciduous trees in fall and winter) on soil microbial biomass (data from repeated measurements), we used a linear mixed‐effects model. We tested the interaction of plot identity (species identity of monocultures) and season [spring (March, April, May), summer (June, July, August), fall (September, October, November), and winter (December, January, February)]. The model included plot nested in experimental block, Machine ID, and soil water content as random effects.

## 3 RESULTS

### 3.1 Wood decomposition

**Figure 2:**
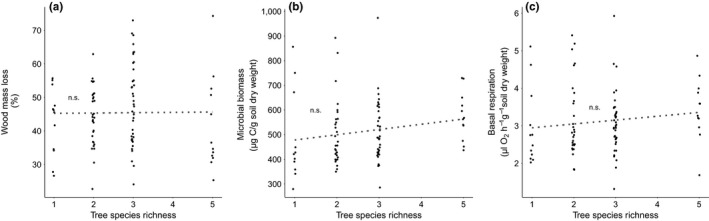
Wood mass loss (a), soil microbial biomass (b), and soil basal respiration (c) as affected by tree species richness (1, 2, 3, and 5 species). Dotted lines show the nonsignificant trend

The average percent wood mass loss determined during the study period ranged from 42.1 ± 8.8% (mean ± *SD*) in 5‐species mixtures to 48 ± 12.4% in 3‐species mixtures. The data varied strongly among plots ranging from 22.8 ± 3.2 to 74.4 ± 23.9%. Tree species richness did not explain a significant proportion of the variation in wood mass loss (Figures 2a and S1, Table 1a).

**Table 1:** Linear mixed‐effects (LME) model table of chi‐square and *p*‐values of tested fixed effects of the tested LME models. (a) Wood mass loss. Fixed effects after model selection are tree species richness (TSR), presence of pine, presence of spruce, and interactions on wood mass loss. (b) Soil microbial biomass (November). Fixed effects after model selection are: TSR, presence of beech, presence of oak, presence of pine, presence of spruce, and interaction effects on soil microbial biomass (c) Soil microbial biomass (Time series). Fixed effects are season, monoculture plot identity, and their interaction on average soil microbial biomass. (d) Soil basal respiration. Fixed effects after model selection are TSR, presence of pine, presence of spruce, and interaction effects on soil basal respiration. ↑: significant positive effect, ↓: significant negative effect. Significant fixed effects (*p* < .05) are shown bold

	*χ* ^2^	*p*	
(a) Wood mass loss			
TSR	0.14	.7043	
**Pine**	10.85	**.0010**	↑
**Spruce**	6.20	**.0128**	↓
TSR:Pine	0.34	.5607	
**TSR:Spruce**	5.05	**.0247**	
**Pine:Spruce**	5.82	**.0158**	
(b) Soil microbial biomass (November)			
TSR	1.50	.2205	
**Beech**	4.91	**.0267**	↓
**Oak**	3.99	**.0459**	↓
**Pine**	6.77	**.0093**	↑
**Spruce**	10.85	**.0001**	↑
**TSR:Beech**	3.93	**.0473**	
Bech:Pine	2.01	.1565	
Oak:Pine	1.69	.1939	
Oak:Spruce	1.48	.2235	
(c) Soil microbial biomass (Time series)			
**Season**	8.70	**.0335**	
**Plot identity**	31.28	**.0001**	
**Season:Plot identity**	42.57	**.0001**	
(d) Soil basal respiration (November)			
TSR	2.62	.1054	
**Pine**	10.19	**.0014**	↑
**Spruce**	21.65	**.0001**	↑
Spruce:Pine	1.05	.3048	
TSR:Pine	0.53	.4675	

**Figure 3:**
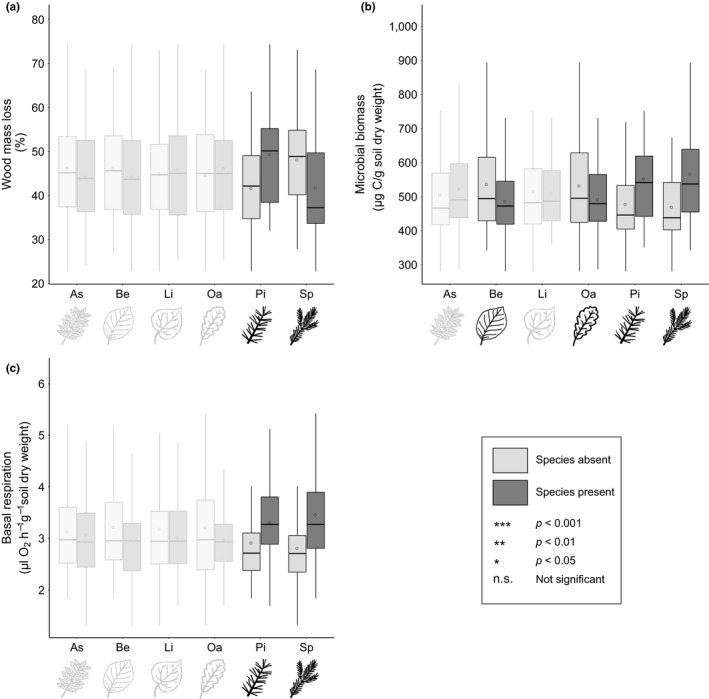
Wood mass loss (a), soil microbial biomass (b), and soil basal respiration (c) as affected by the presence (dark gray) or absence (light gray) of ash (As), beech (Be), lime (Li), oak (Oa), pine (Pi), and spruce (Sp). Black dots show the average values among all plots. ***: *p* < .001; **: *p* < .01; *: *p* < .05; n.s.: not significant. Nonsignificant results are grayed out

However, the presence of certain tree species within the plots significantly influenced wood mass loss (Table 1a). While the presence of pine significantly increased wood decomposition, the presence of the other conifer species, spruce, significantly decreased wood decomposition. Ash and beech tended to decrease wood mass loss, while the presence of lime and oak tended to increase wood mass loss (Figure 3a), but none of these effects were statistically significant.

**Figure 4:**
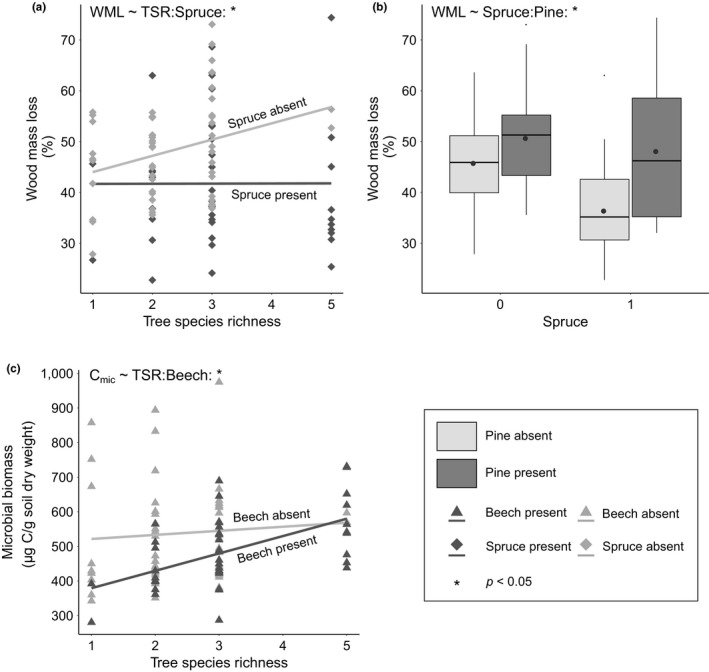
Belowground ecosystem functions as affected by various interactions. (a) Interaction between tree species richness (1, 2, 3, and 5 species) and the presence of spruce on wood mass loss. (b) Interaction between the presence of spruce and pine on wood mass loss. Black dots indicate average wood mass loss. (c) Interaction between tree species richness (1, 2, 3, and 5 species) and the presence of beech on soil microbial biomass

We found a significant interaction effect between tree species richness and the presence of spruce, where wood mass loss increased with tree species richness in the absence of spruce, while it was unaffected by tree species richness in the presence of spruce (Figures 4a and S1, Table 1a). Furthermore, the presence of spruce and the presence of pine had a significant interaction effect on wood mass loss, indicating the positive effect of pine on wood mass loss was more pronounced in the absence of spruce than in its presence (Figures 4b and S1).

### 3.2 Soil microbial biomass

Soil microbial biomass ranged from 280.5 µg  C g^−1^ soil dry weight in a monoculture of beech to 974.9 µg  C g^−1^ soil dry weight in a 3‐species mixture (*Fraxinus excelsior*/*Picea abies*/*Pinus sylvestris*) (overall mean: 513.7 ± 129.3 µg  C g^−1^ soil dry weight). Soil microbial biomass tended to increase with increasing tree species richness, but the effect was not statistically significant (Figures 2b and S2, Table 1b). However, there was a significant effect of tree species identity. While the presence of pine and the presence of spruce significantly increased soil microbial biomass, the presence of beech as well as the presence of oak significantly decreased soil microbial biomass (Table 1b). Ash and lime did not significantly affect soil microbial biomass (Figure 3b). Moreover, we found a significant interaction effect between the presence of beech and tree species richness for soil microbial biomass, indicating that soil microbial biomass increased with increasing tree species richness in the presence of beech, while this relationship was not significant in the absence of beech (Figure 4c, Table 1b).

For the averaged soil microbial biomass data, we found a significant interaction effect between monoculture plot identity (tree species in monoculture) and season (i.e., spring, summer, fall, and winter), indicating that the seasonal change in soil microbial biomass depends on the identity of the present tree species (Table 1c). Soil microbial biomass in spruce (significantly) and pine (by trend) plots was higher during winter, whereas most other combinations tended to have lower microbial biomass (Figure S3).

### 3.3 Relationship between wood mass loss and soil microbial biomass

**Figure 5:**
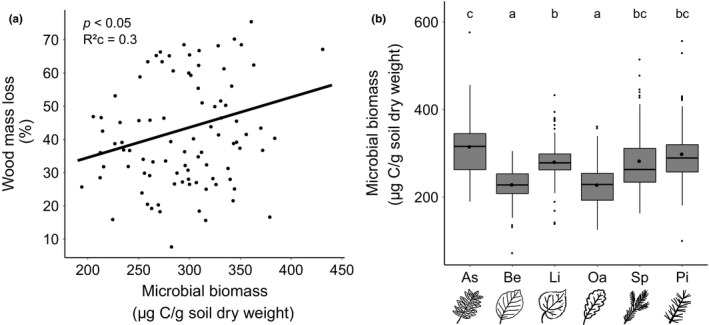
Relationship between soil microbial biomass and wood mass loss, as well as tree identity. (a) Significant positive relationship between average soil microbial biomass from August 2016 to October 2017 and wood mass loss. (b) Significant effect of plot identity (monoculture species; ***: *p* < .001) on average soil microbial biomass from August 2016 to October 2017. Black circles show average values per plot identity. Different letters denote significant differences (Tukey's range test)

In addition to microbial data from an endpoint sampling, we also analyzed average data from monoculture and 5‐species mixture plots that were taken between August 2016 and October 2017 and found a significant positive relationship between wood mass loss and soil microbial biomass (Figure 5a). Moreover, averaged soil microbial biomass varied significantly among monocultures, with the highest values in ash monocultures, intermediate levels in lime, pine, and spruce monocultures, and the lowest values in beech and oak plots (Figure 5b).

### 3.4 Soil basal respiration

Soil basal respiration ranged from 1.3 µl O_2_ hr^−1^ g^−1^ soil dry weight in a 3‐species mixture plot (*Fagus sylvatica*/*Fraxinus excelsior*/*Pinus sylvestris*) to 5.9 µl O_2_ hr^−1^ g^−1^ soil dry weight in another 3‐species mixtures plot (*Fraxinus excelsior*/*Picea abies*/*Pinus sylvestris*) (overall mean: 3.1 ± 0.9 µl O_2_ hr^−1^ g^−1^ soil dry weight). Soil basal respiration tended to increase with tree species richness, but the effect was not statistically significant (Figures 2c and S4, Table 1d). In plots with spruce or pine, there was a significant increase in basal respiration, while the presence of ash showed no significant effect. The presence of beech, lime, and oak tended to decrease soil basal respiration, but these effects were not significant (Figure 3c, Table 1d).

### 3.5 Carbon‐to‐nitrogen ratio of leaf litter

**Figure 6:**
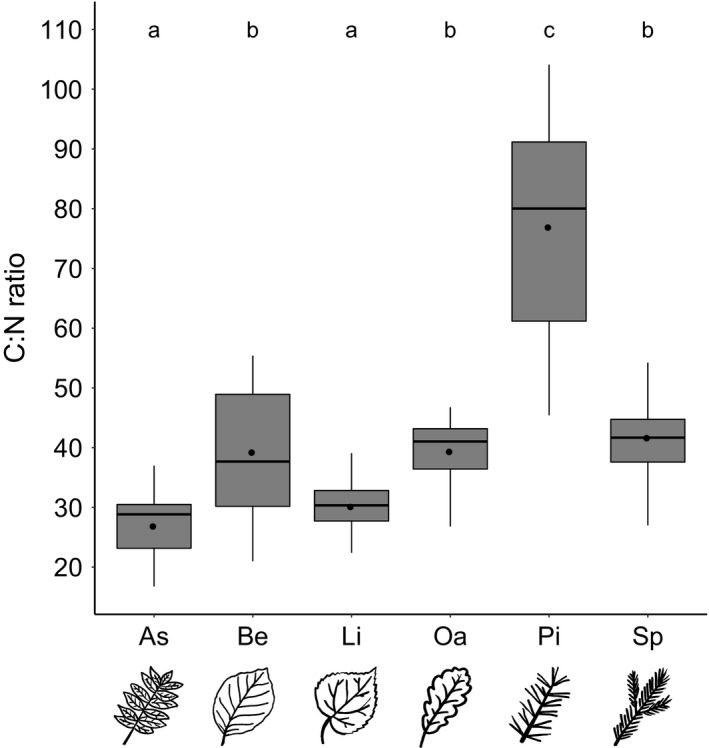
Litter C:N ratio of the studied tree species. Black circles show average values per tree species. Different letters denote significant differences (Tukey's range test)

The litter C:N ratio of the different tree species in the Kreinitz experiment differed significantly (*χ* ^2^ = 563.07; *p* < .001; *df* = 5, 146). Ash and lime had the lowest average C:N ratio and pine the highest. Beech, oak, and spruce ranged in between (Figure 6).

### 3.6 Tree identity effects on soil surface temperature

**Figure 7:**
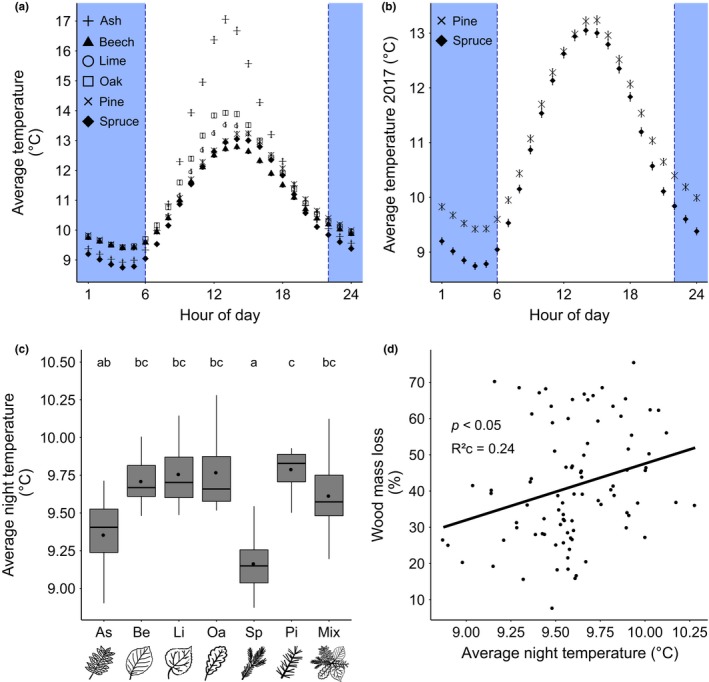
Daily average temperature of monoculture plots. (a) Plot‐level average temperature per hour of day from February 2017 to December 2017 for all monocultures. Blue areas indicate defined period without direct sunlight. Values are average values over all measuring days (*n* = 312). (b) Average soil surface temperature per hour of day from February 2017 to December 2017 in spruce and pine monoculture plots. Blue areas indicate defined period without direct sunlight. Values are means ± *SE* over all measuring days. (c) Tree identity effect (***: *p* < .001) (monocultures and 5 species mixtures) on average night soil surface temperature from February 2017 to December 2017. Black circles show average values per monoculture respectively 5‐species mixture. Different letters denote significant differences (Tukey's range test). (d) Positive relationship (*p* < .05) between average night soil surface temperature from February 2017 to December 2017 and wood mass loss

The here presented temperature data were derived from all present monocultures (six tree species with two replicates each; *n* = 12 plots) and 5‐species mixtures (*n* = 12 plots). The average overall temperature varied from 10.71 ± 5.90°C on spruce monocultures to 11.84 ± 7.38°C on ash monocultures (Table 2). During a 24‐hr cycle, the average soil surface temperature on all plots followed the expected diurnal variation with strong differences among the tree species between 10 p.m. and 6 a.m. and similar values during expected hours of sunlight (Figure 7a,b).

**Table 2:** Average temperature + *SD* and average night temperature [between 10 p.m. (CET) and 6 a.m. (CET)] + *SD* in 2017 on monocultures and 5‐species mixtures

	Ash	Beech	Lime	Oak	Pine	Spruce	5‐species mixture
24‐hr average temperature	11.84 ± 7.38	10.87 ± 5.54	11.15 ± 5.82	11.30 ± 5.90	11.10 ± 5.72	10.71 ± 5.90	10.88 ± 5.75
Average night temperature	9.35 ± 5.46	9.71 ± 4.95	9.75 ± 5.12	9.76 ± 5.14	9.79 ± 4.92	9.16 ± 5.07	9.59 ± 5.06

The tree community treatment had a significant effect on the average night temperature (Figure 7c). The average soil surface temperature during the night hours varied from 9.16 ± 5.07°C on spruce monocultures to 9.79 ± 4.92°C on pine monocultures (Table 2, Figure 7c). We found the highest difference in average night temperature (Δ*T*_N_ = 0.63°C) between the pine and spruce monocultures (Figure 7c), which also differed the most in wood mass loss (Figure 3a, Table 1a). The subsequent regression analysis revealed a significant positive relationship between average night temperature on wood mass loss (Figure 7d).

## 4 DISCUSSION

We studied the effects of tree species richness and identity on wood decomposition in a 12‐year‐old tree diversity experiment and tested the mediating effects of microclimatic conditions and soil microbial biomass as potential explanatory mechanisms. Our study suggests that tree species richness alone is not sufficient to explain wood decomposition in the studied young temperate forest stand. Instead, we found evidence that wood decomposition depends on tree identity‐induced changes in soil surface temperature and soil microbial biomass.

Following the existing evidence for positive relationships between biodiversity and ecosystem functions (Bardgett & Van Der Putten, 2014; Hooper et al., 2000; Huang et al., 2018; Tilman, Isbell, & Cowles, 2014), we expected to find enhanced wood decomposition, soil basal respiration, and microbial biomass with higher tree species richness. Contrary to this expectation, we could not reveal any effects solely driven by tree species richness. The reason for this lack of evidence for clear tree species richness effects on soil microbial properties and wood decomposition may lay in the context dependency of effects of different diversity facets. For instance, there is empirical evidence that complementarity effects with increased tree species richness are stronger at nutrient‐poor sites than at nutrient‐rich sites (Paquette & Messier, 2011). Thus, the fact that the Kreinitz experiment was established on a former nutrient‐rich arable land may have limited significant tree species richness effects on soil properties. While the Kreinitz experiment is among the oldest tree diversity experiments in Europe (12 years at the time of sampling), it still has to be considered a young stand. This may have further limited the significance of biodiversity effects in our study, as plant diversity effects on ecosystem functioning have been shown to increase over time in experimental grasslands and forests (Guerrero‐Ramírez et al., 2017), and soil responses to variations in plant diversity may need some time to materialize (Eisenhauer, Reich, & Scheu, 2012; Thakur et al., 2015). Moreover, the fact that these rather young experiments have an even age distribution among tree individuals may further limit complementarity effects among individuals and species, which may contribute to weak tree species richness effects (Leuschner, Jungkunst, & Fleck, 2009).

However, providing some support for our hypothesis (i), we observed significant interaction effects, where the tree species richness effect on soil microbial biomass and wood mass loss depended on the presence of beech and spruce, respectively. In the presence of beech, soil microbial biomass increased with increasing tree species richness, but not in its absence. The second significant interaction suggests that the presence of spruce canceled out a tree species richness effect on wood mass loss. Only in the absence of spruce, tree species richness increased wood mass loss. Considering the evidence we found for a negative effect of the presence beech on soil microbial biomass over time, we suggest that the interaction effect with tree species richness was actually driven by a dilution effect (Baeten et al., 2013). Due to equal tree density across plots, the proportion of beech trees within a plot decreases with increasing tree species richness, and therefore, its effect on soil microbial properties is expected to get weaker. While the exact mechanism behind the canceling result of spruce remains unclear, it also provides further support for the observation that other biodiversity facets—such as functional trait identity and diversity—may be crucial to understand relationships between tree diversity and soil ecosystem functions in general (Cesarz et al., 2013; Craven et al., 2018; Schuldt et al., 2019). More specifically, recent studies have shown that certain plant traits are especially relevant for wood decomposition, suggesting that the identity of trees can be of particular significance (Fujii et al., 2016; Jewell et al., 2017; Joly et al., 2017). We considered the role of tree identity for the interactions and the present evidence from other studies in our further analysis and could confirm the relevance of tree identity for decomposition processes and soil microbial properties in forests. We found evidence that the presence of pine increased wood decomposition, while the presence of spruce led to a strong decline. When both tree species were present, the positive pine effect was considerably weakened. This strong negative effect of spruce on wood mass loss may also play a role in the inhibition of tree species richness effects on wood mass loss described above.

We also found a significant positive effect of the presence of both coniferous trees on microbial biomass and soil basal respiration, while the presence of oak and beech decreased soil microbial properties. Those findings were surprising, since other studies showed a negative effect of evergreen tree species on decomposition (Joly et al., 2017), as well as on soil microbial properties (Vesterdal, Elberling, Christiansen, Callesen, & Schmidt, 2012), mainly due to poor litter quality (Ayres et al., 2009; Scheibe et al., 2015). Indeed, we also found that specifically the pine litter in Kreinitz had a very high C:N value compared to the other species present, indicating poor litter quality. This counterintuitive combination of poor litter quality and positive influence on decomposition and soil microbial properties may be explained by the substrate quality–matrix quality interaction hypothesis by Freschet, Aerts, and Cornelissen (2012). They state that decomposition rates of recalcitrant plant material (such as wood) are higher in a matrix of similar quality (such as pine litter) than in a matrix of higher quality. Considering this, our hypothesis (ii) was partly confirmed. While we did find evidence for a tree identity effect via litter quality, the effects were opposite to what we had expected.

We also have to consider a seasonal effect for the results of the sampling in November (late fall). While deciduous tree species withdraw nutrients and chlorophyll from leaves before winter, reducing their photosynthesis and overall activity (Givnish, 2002), coniferous tree species are evergreen and sustain a higher activity and may thus have higher rates of rhizodeposition, fueling soil communities (Högberg et al., 2001). Further analysis of our time series data revealed a significant interaction effect between the identity of monocultures and season influencing soil microbial biomass. Specifically both coniferous tree species increased soil microbial biomass during the winter months. A finding  that matches to the specific phenologic traits of spruce and pine. These results further underline the necessity to consider year‐round effects of tree community composition on ecosystem functions. It is also known that wood decomposition in forests is mainly driven by fungal biomass (Baldrian et al., 2012; Eastwood et al., 2011).

An increased fungal biomass is often associated with conifers and pine in particular (Eastwood et al., 2011; Ushio, Balser, & Kitayama, 2013; Zechmeister‐Boltenstern, Michel, & Pfeffer, 2011). The increased microbial biomass in the presence of pine may thus be explained by a higher abundance of soil fungi in relation to bacteria. However, we can only speculate about potential differences in soil microbial communities as the methods we applied in the present study do not provide any information on community composition. Future studies should explore soil microbial and detritivore communities in more detail.

At this point, the negative effect of the presence of spruce on wood mass loss remains unexplained and indicates additional mechanisms. Several secondary metabolites of conifer litter, such as phenols and tannins (Kanerva & Smolander, 2008), are known to inhibit decomposition and soil microbial properties (Ushio et al., 2013). However, this assumption was not supported by our data on soil microbial properties, which were increased instead of decreased in the presence of spruce. Further chemical analysis of secondary metabolites in the leave litter of the Kreinitz experiment could help to explore this possible mechanism.

In addition to chemical litter properties and the soil community composition, decomposition is also governed by local environmental conditions like temperature (Harmon et al., 2004; Pietsch et al., 2014), which was shown to depend on the presence of particular plant species and their functional identity (Eviner & Chapin III, 2003; Martius et al., 2004). Recent studies presented more evidence for a connection between tree species identity, microclimatic conditions, and specifically wood decomposition in temperate and subtropical forests (Joly et al., 2017; Pietsch et al., 2018). Despite those previous findings, we could not explain the reported differences in wood decomposition rates for the presence of spruce and the presence of pine using the 24‐hr average soil surface temperature during the study period. However, further analysis of our temperature data revealed a more context‐dependent relationship: We found a significant tree identity effect on the daily temperature average and a strong difference in temperature holding capacities of spruce and pine monocultures over the course of the day. During noon and shortly before and after, both (spruce and pine) monoculture stands showed similar soil surface temperatures. At night, however, they differed significantly: The average soil surface temperature during hours without sunlight was significantly lower in spruce plots than that in pine plots. Those results suggest that the plots with spruce lose the temperature gained during hours of sunlight much faster than pine monocultures that maintain higher temperature. The underlying reason for this is most likely the different structures of pine and spruce litter, that is, of the surrounding structure of the decomposing wooden sticks. In pine monocultures, the accumulated litter formed a thick and entangled layer of needles that may have insulated the soil surface against temperature fluctuations. The spruce litter layer was comparably thin and loose, likely resulting in a faster temperature loss of the soil surface.

Based on this insight, we used the average night (hours without direct sunlight) instead of daily soil surface temperatures for our analyses and observed a significant positive relationship with wood mass loss in line with current knowledge and confirming our hypothesis (iv). Moreover, this microclimate effect may explain the opposing responses of increased microbial biomass but decreased wood mass loss on plots containing spruce, despite our finding of increased wood mass loss with increased soil microbial biomass [confirming hypothesis (iii)]. Both spruce and pine seem to increase soil microbial biomass (e.g., *via* resource availability and chemical litter composition), but the positive effect of this increased biomass on wood decomposition is likely to be influenced by a longer period with higher temperature, stimulating the activity of the soil microbial biomass (Harmon et al., 2004; Weedon et al., 2009).

## 5 CONCLUSION

We conclude that wood decomposition in temperate forests strongly depends on tree species identity. Consequently, future tree diversity experiments should consider the role of different biodiversity facets, such as tree identity, different tree traits, their functional diversity (Craven et al., 2018; Schuldt et al., 2019), and their abiotic and biotic effects. Our study adds to the body of literature highlighting the significant role of microclimatic conditions, such as surface temperature, on decomposition processes in forests. Furthermore, the insight of the importance of night temperature compared to overall daily temperature adds information on the context dependency to this relationship. To improve our understanding of the relationship between tree identity, diversity, and the soil microbiome, we suggest to further investigate the microbial community composition (Lange et al., 2015), starting with analyses concerning the proportion and activity of soil fungi that are related to decomposition processes. This could go hand in hand with more specific time series analyses to explore the temporal dynamics of biotic and abiotic drivers of decomposition (Eisenhauer et al., 2018). In the context of climate change, it would be of particular interest to further study the potential role of the litter layer to affect or maybe even buffer soil and soil surface temperatures under warming conditions.

## CONFLICT OF INTEREST

None declared.

## AUTHORS' CONTRIBUTIONS

All authors approved the final version of the manuscript and agreed to be accountable for all aspects of the work in ensuring that questions related to the accuracy or integrity of any part of the work are appropriately investigated and resolved. Felix Gottschall contributed substantially to the conception and design of the experiment and to the acquisition, analysis, and interpretation of data for the work. Furthermore, he drafted the work. Till E. Newiger‐Dous contributed substantially to the acquisition and interpretation of data and the critical revision for important intellectual content of the work. Sophie Davids contributed substantially to the acquisition and interpretation of data and the critical revision for important intellectual content of the work. Dr. Harald Auge contributed substantially to the conception and design of the work, as well as to the analysis of the data. He critically revised the work for important intellectual content. Dr. Simone Cesarz contributed substantially to the conception and design of the work, as well as to the analysis and interpretation of the data. She critically revised the work for important intellectual content. Dr. Nico Eisenhauer contributed substantially to the conception and design of the work, as well as to the analysis and interpretation of the data.  He critically revised the work for important intellectual content.

## Data Availability

The data of this work are deposited on the Dryad Digital Repository (DOI: https://doi.org/10.5061/dryad.m3s4t57), following the data accessibility guidelines of Ecology and Evolution.
