# Supplementary material for: Tree species identity determines wood decomposition via microclimatic effects
Source: Ecol Evol. 2019 Sep 27;9(21):12113–27. doi: 10.1002/ece3.5665 (PMC6854332; doi:10.1002/ece3.5665)
Supplement: Supplementary file 1 [file ECE3-9-12113-s001.docx]

Supporting information:

Tree species identity determines wood decomposition via microclimatic effects

Felix Gottschall^1,2,*^, Sophie Davids^1,2^, Till E. Newiger-Dous^1,2^, Harald Auge^1,3^, Simone Cesarz^1,2,§^, Nico Eisenhauer^1,2,§^

^1^ *German Centre for Integrative Biodiversity Research (iDiv) Halle-Jena-Leipzig, Deutscher Platz 5e, 04103, Leipzig, Germany*

*^2^   Institute of Biology, Leipzig University, Deutscher Platz 5e, 04103, Leipzig, Germany*

*^3^   Department of Community Ecology, Helmholtz-Centre for Environmental Research – UFZ, Theodor-Lieser-Str. 4, 06120, Halle, Germany*

*^*^   Correspondence to: Felix Gottschall, German Centre for Integrative Biodiversity Research (iDiv) Halle-Jena-Leipzig, Deutscher Platz 5e, 04103, Leipzig, Germany felix.gottschall@idiv.de*

*^§^  shared senior authorship*


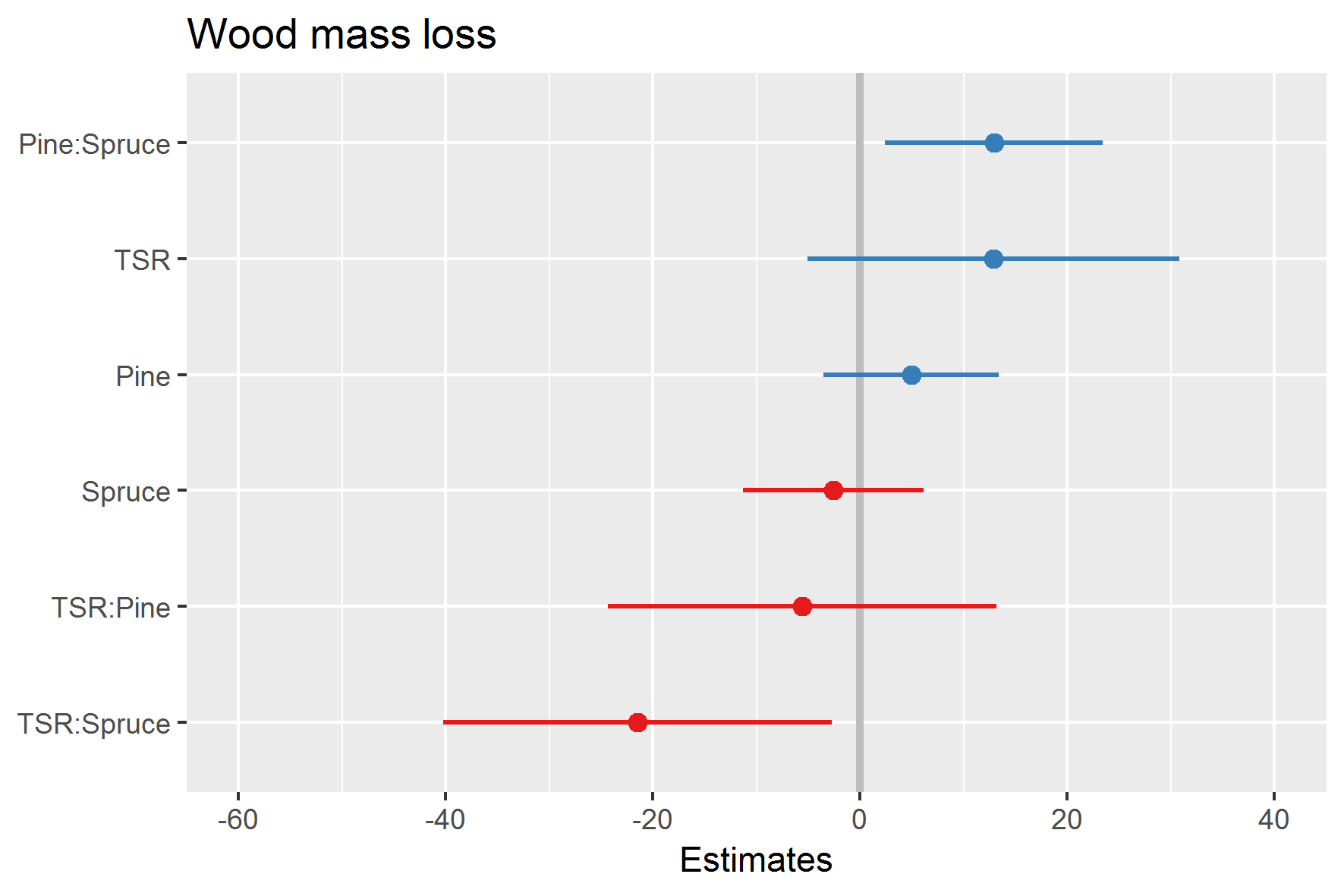


**Fig. S1.** Forest plot showing the effect sizes and 95% confidence intervals for the individual fixed effects and interactions for the linear mixed effects model testing the effect of tree species identity (presence of pine and spruce) and tree species richness (TSR, as continuous variable) on wood decomposition (i.e. mood mass loss). The grey line denotes a mean of 0.


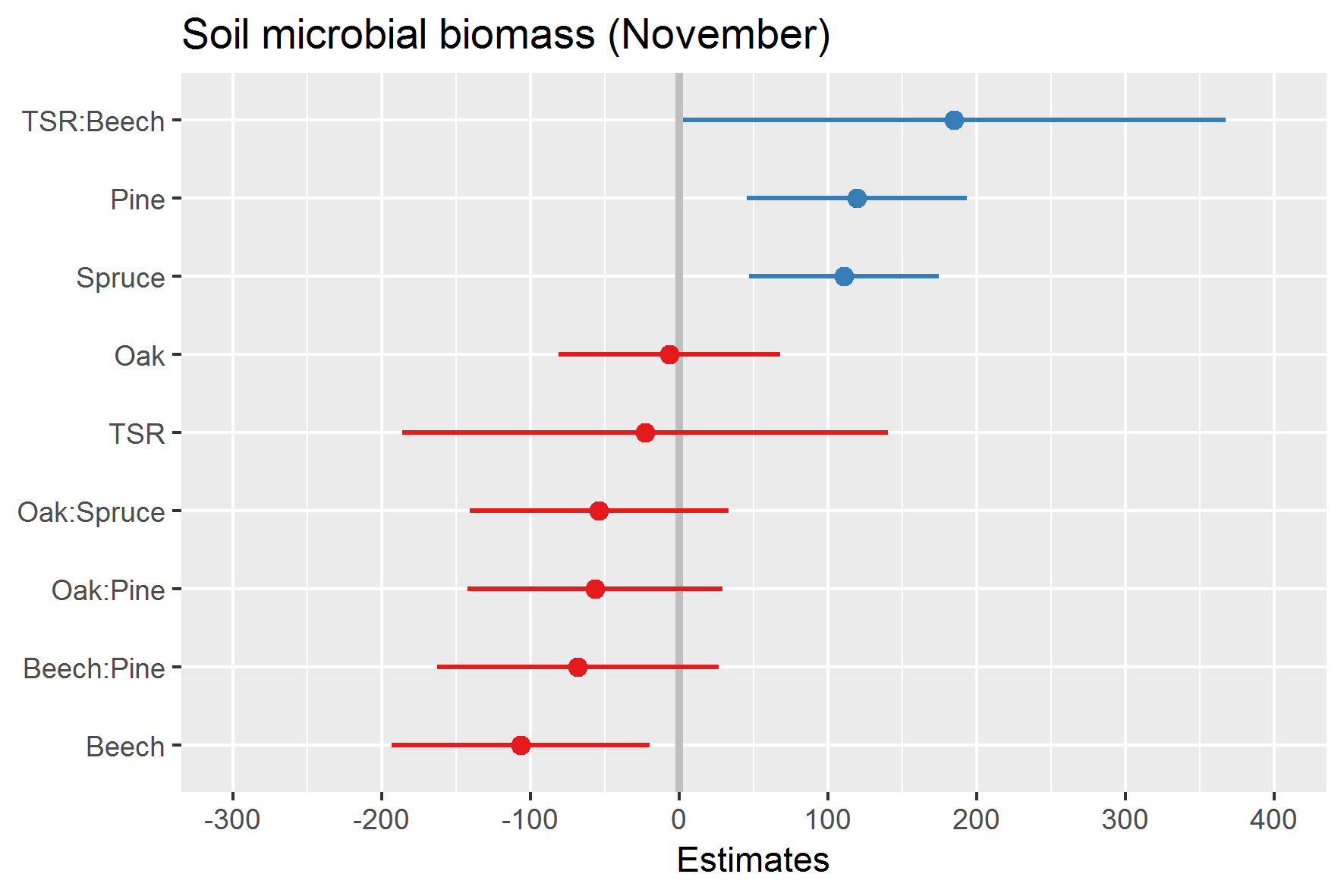


**Fig. S2.** Forest plot showing the effect sizes and 95% confidence intervals for the individual fixed effects and interactions for the linear mixed effects model testing the effect of tree species identity (presence of beech, oak, pine, and spruce) and tree species richness (TSR, as continuous variable) on soil microbial biomass in November 2017. The grey line denotes a mean of 0.


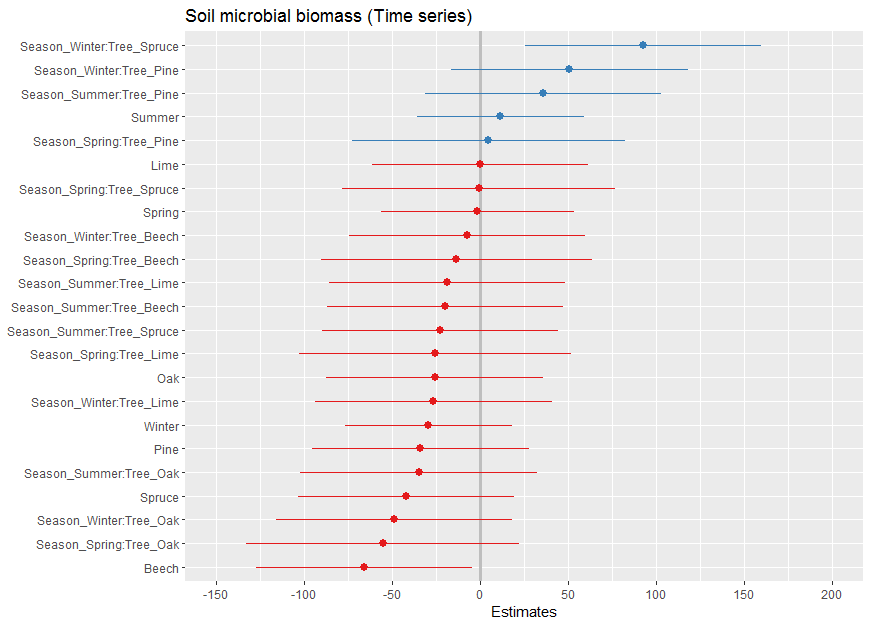


**Fig. S3.** Forest plot showing the effect sizes and 95% confidence intervals for the individual fixed effects and interactions for the linear mixed effects model testing the effect of plot identity (species identity of monocultures) and season (spring (March, April, May), summer (June, July, August), fall (September, October, November), and winter (December, January, February)) on soil microbial biomass during the decomposition experiment. The grey line denotes a mean of 0.


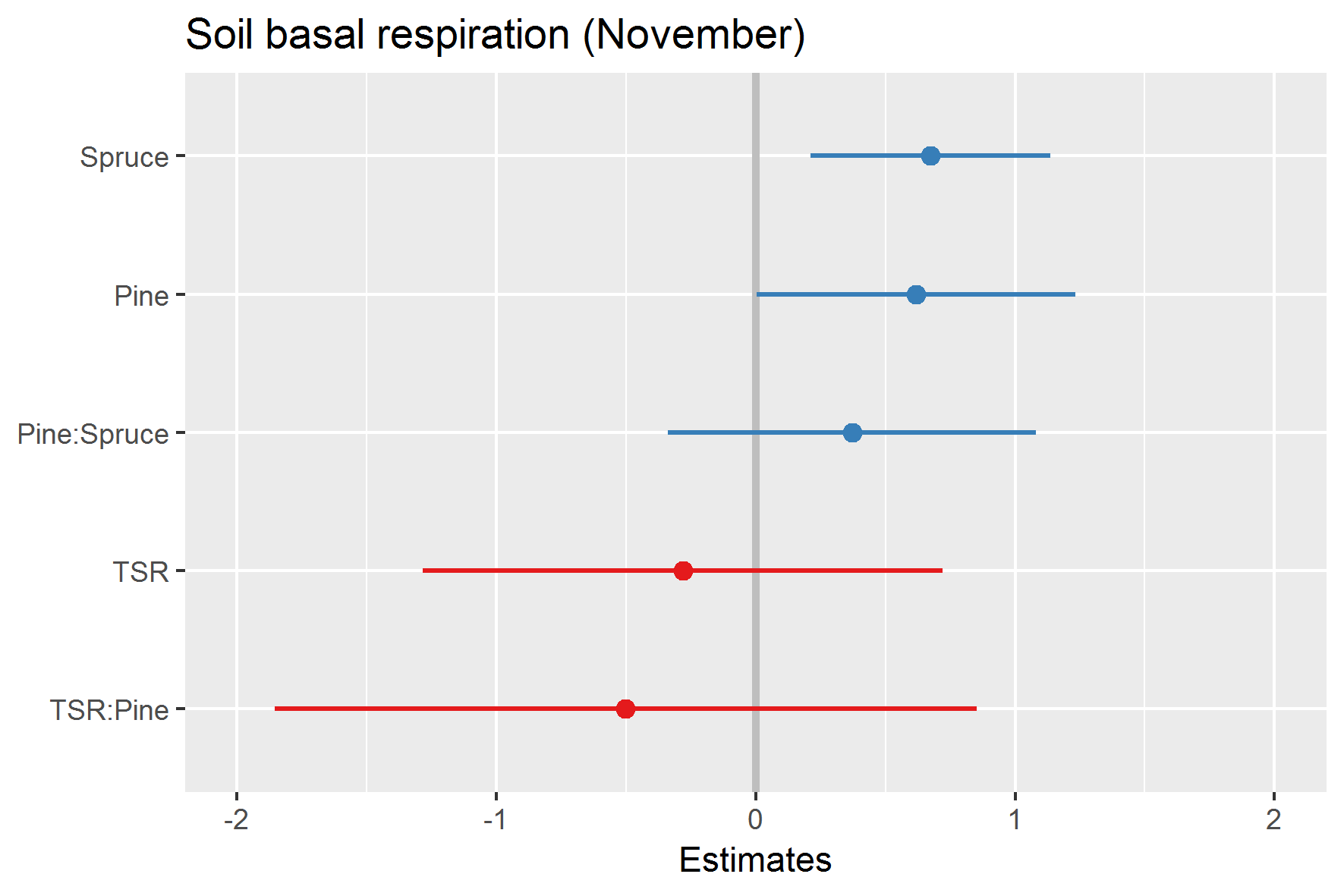


**Fig. S4.** Forest plot showing the effect sizes and 95% confidence intervals for the individual fixed effects and interactions for the linear mixed effects model testing the effect of tree species identity (presence of pine and spruce) and tree species richness (TSR, as continuous variable) on soil basal respiration in November 2017. The grey line denotes a mean of 0.
